# Electrochemical detection of dopamine using periodic cylindrical gold nanoelectrode arrays

**DOI:** 10.1038/s41598-018-32477-0

**Published:** 2018-09-19

**Authors:** Da-Seul Kim, Ee-Seul Kang, Seungho Baek, Sung-Sik Choo, Yong-Ho Chung, Donghyun Lee, Junhong Min, Tae-Hyung Kim

**Affiliations:** 1 School of Integrative Engineering, Chung-Ang University, 84 Heukseok-ro, Dongjak-gu, Seoul, 06974 Republic of Korea; 2 Department of Chemical Engineering, Hoseo University, Asan City, Chungnam 31499 Republic of Korea; 3 Integrative Research Center for Two-Dimensional Functional Materials, Institute of Interdisciplinary Convergence Research, Chung-Ang University, Seoul, 06974 Republic of Korea

## Abstract

Dopamine is a key molecule in neurotransmission and has been known to be responsible for several neurological diseases. Hence, its sensitive and selective detection is important for the early diagnosis of diseases related to abnormal levels of dopamine. In this study, we reported a new cylindrical gold nanoelectrode (CAuNE) platform fabricated via sequential laser interference lithography and electrochemical deposition. Among the fabricated electrodes, CAuNEs with a diameter of 700 nm, 150 s deposited, was found to be the best for electrochemical dopamine detection. According to cyclic voltammetry results, the linear range of the CAuNE-700 nm was 1–100 µM of dopamine with a limit of detection (LOD) of 5.83 µM. Moreover, owing to the homogeneous periodic features of CAuNEs, human neural cells were successfully cultured and maintained for more than 5 days *in vitro* without the use of any extracellular matrix proteins and dopamine was detectable in the presence of these cells on the electrode. Therefore, we concluded that the developed dopamine sensing platform CAuNE can be used for many applications including early diagnosis of neurological diseases; function tests of dopaminergic neurons derived from various stem cell sources; and toxicity assessments of drugs, chemicals, and nanomaterials on human neuronal cells.

## Introduction

Dopamine is one of the most researched neurotransmitters because of its important functions in the human body (e.g., human metabolism, cardiovascular, central nervous, renal, and hormonal systems)^1–7^. Since dopamine is critical for signal transmissions to the brain, an inadequate level of dopamine can lead to many neurological diseases/disorders such as schizophrenia, attention deficit hyperactivity disorder (ADHD) and Parkinson’s disease (PD)^8–14^.

To address this issue, there is an urgent need to develop platforms for the sensitive and selective detection of dopamine concentrations. To date, conventional colorimetric/column-based methods (e.g., ELISA, HPLC) have been reported to be effective for the detection of dopamine. However, these techniques are generally laborious and time consuming, which hinders the early diagnosis of several representative dopamine-related neurological diseases, and require several expensive analytical reagents^15–19^. Electrochemical detection is a simple and convenient technique and has therefore attracted increasing interest for measurement of various neurotransmitters including dopamine, gamma-aminobutyric acid, and serotonin^20–24^. Unlike other biological molecules such as glucose, dopamine is highly redox active and can effectively be measured without the use of other redox couples and/or enzymes required to facilitate the reduction/oxidation of the target molecules^19,25–30^. However, one critical concern for the detection of dopamine in blood is its low concentration level^1,5^, which is further amplified by the signal interference from other biological molecules such as glucose, ascorbic acid (AA), and uric acid (UA). To this end, numerous studies have reported a variety of platforms by mostly integrating conductive micro-/nanomaterials or polymers on the surface of electrodes^30^. Owing to the high conductivity and excellent electrocatalytic properties, metals such as gold, silver, and platinum have been modified on the electrode surface, mostly in the form of nanoparticles/nanostructures, to take advantage of their high surface-to-volume ratios. The methods employed to modify these metallic nanostructures on the conducting substrates can be categorized into two groups: (i) synthesis of metal nanoparticles and their following attachments onto the electrode surface via chemical linkers and (ii) direct formation of metal nanostructures on the substrates via electrochemical/chemical deposition. Nanostructured electrodes integrated into sensing platforms have been found to be highly effective to enhance both sensitivity and selectivity not only toward neurotransmitter detection including dopamine but also toward other biological materials (e.g., DNA, RNA, proteins, pathogens, glucose). However, comparing these nanostructured electrodes with conventional products, critical concerns have been raised, especially the instability of the electrical signals and the increase in the signal variations due to the difficulty in controlling the number, size, and shape of nanostructures modified on the electrode surfaces.

**Figure 1:**
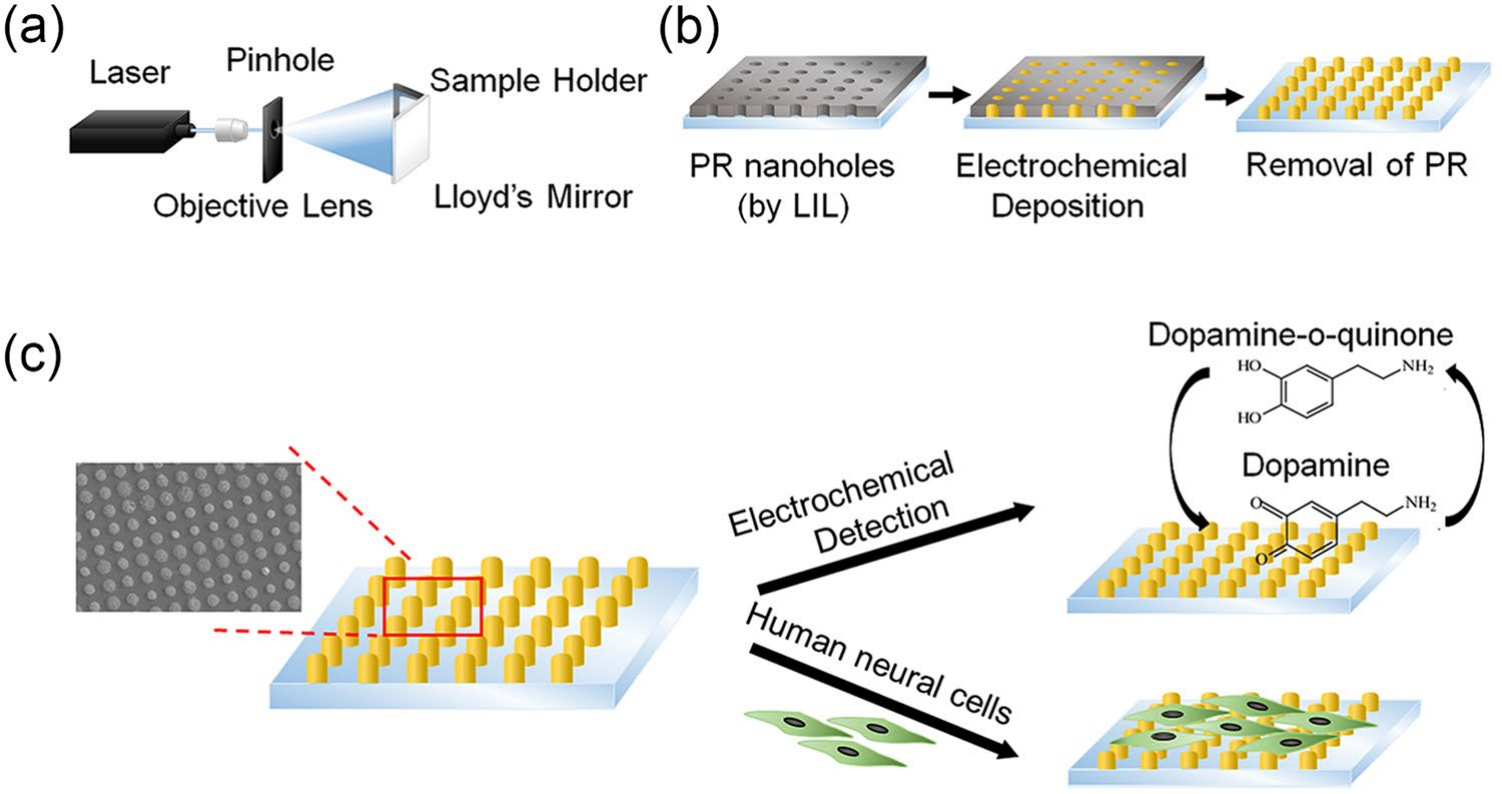
Schematic representing the strategy of electrochemical signal detection of dopamine using CAuNE platform. (**a**) Lloyd’s mirror interferometer for LIL. UV light from the laser passes through the objective lens and through the pinhole containing a spatial filter. (**b**) Sequential steps of fabrication of CAuNE platform. (**c**) CAuNE platform would be utilized as the substrate for dopamine detection by electrochemistry and cultivation of human neural cells.

In this study, we report a new sensing platform for the effective detection of dopamine based on electrochemical methods. Unlike the previous study reporting the gold nanocup arrays for the detection of dopamine released from rat carcinoma^31^, here, we developed a simple cylindrical gold nanoelectrode with varying its size and height to find the best conditions for measuring both chemical dopamine and the dopamine release from human dopaminergic neurons (SH-SY5Y). Sequential laser interference lithography (LIL) and electrochemical deposition (ECD) were utilized to prepare homogeneous cylindrical gold nanoelectrode (CAuNE) platforms, which are critical to achieve stable dopamine-specific electrochemical signals and to reduce the signal variations (Fig. 1). Using LIL, which eliminates the need for photomask fabrication, homogeneous photoresist (PR) nanoholes of three different diameters (500 nm, 700 nm, 900 nm) were easily fabricated on an indium tin oxide (ITO) surface. The PR nanohole arrays were then used as the templates to fabricate gold nanocylinder arrays via ECD. After confirming the topological characteristics of the CAuNE platforms, the effect of the diameter and height of the nanocylinders on the intensity of the redox signals of dopamine was analyzed by cyclic voltammetry (CV). The CAuNE platform that showed the best performance for dopamine detection was chosen to obtain the linear correlation between electrical signals and dopamine concentration, followed by selectivity testing of the platform toward dopamine using UA and glucose as the interfering molecules^30,32,33^. Since recent studies reported by Bhalla *et al*. have shown that the nanostructured substrates help long-term survival of cells^34,35^, the cell spreading and proliferation of human neural cell line, SH-SY5Y neuroblastoma, on CAuNE platforms were intensively analyzed. This study is incredibly important for developing a new type of biosensing platform that enables early diagnosis of various neurological diseases and evaluates the dopamine production ability of differentiated neuronal cells *ex vivo*.

## Results and Discussions

### Fabrication and characterization of cylindrical gold nanoelectrodes

**Figure 2:**
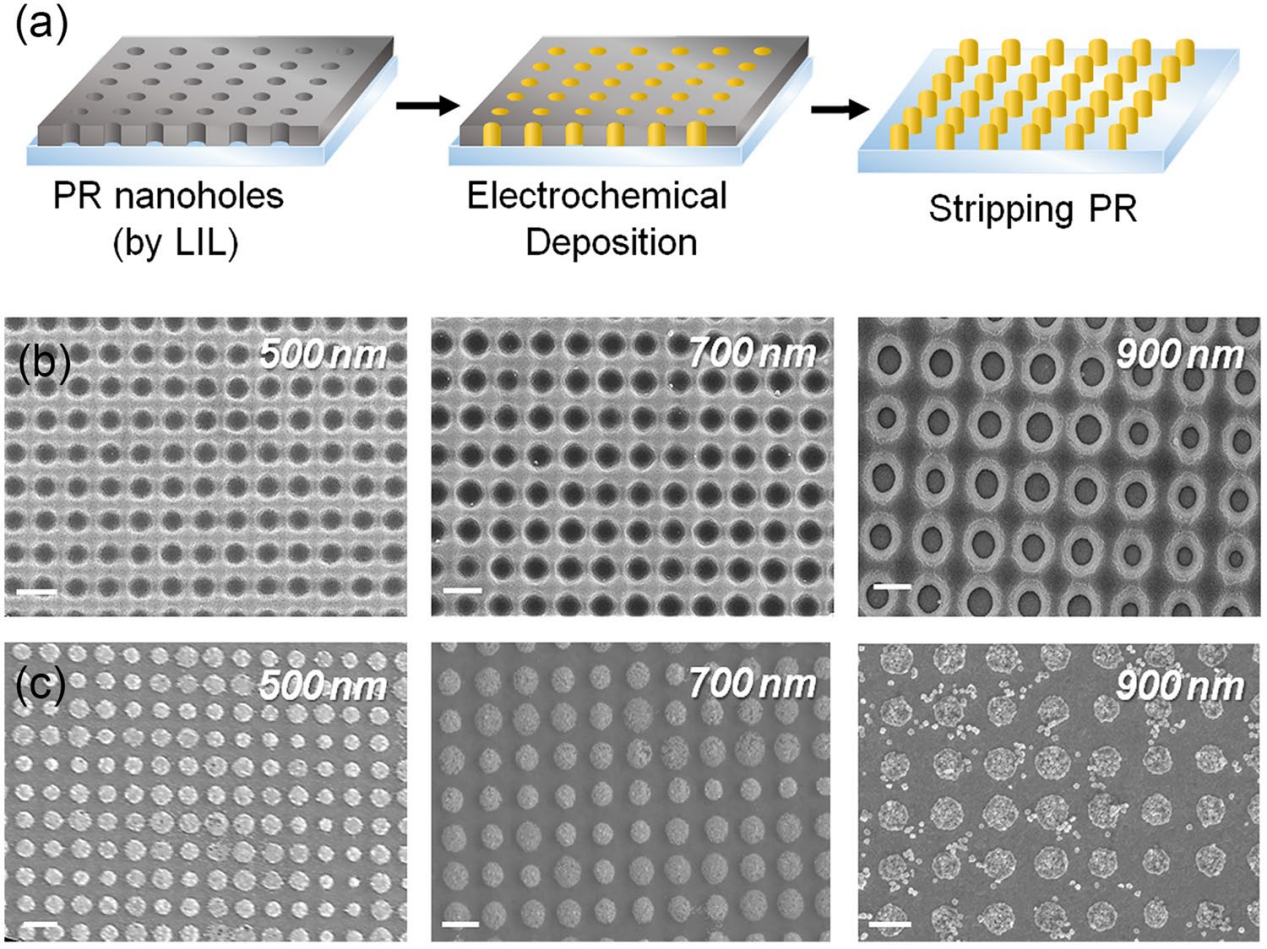
(**a**) Schematic showing sequential steps to fabricate CAuNEs. (**b**) FE-SEM images of three nanopatterns of CAuNEs with diameters of 500 nm, 700 nm, and 900 nm, corresponding to the left illustration of (**a**). (**c**) FE-SEM images of CAuNEs of different diameters after PR stripping step, corresponding to the right illustration of (**a**). Scale bar = 1 µm.

To overcome the challenges of current nanosized platforms, such as high cost, long fabrication time, and huge signal variations^16,18,36,37^, we fabricated a novel sensing platform based on CAuNEs. First, homogenous polymer nanohole patterns were fabricated by LIL, as shown in Fig. 2(a). By varying the incidence angle, that is, the angle between the light incident on the electrode surface and the line perpendicular to the surface, the diameters of the PR nanoholes could be freely varied without the use of a photomask^31,38–45^. The PR nanohole patterns on the ITO substrate were then used as templates to deposit gold via ECD. Compared to other metal deposition methods such as radio frequency magnetron sputtering, chemical vapor deposition, and e-beam evaporation, ECD can easily control the parameters of the nanostructures, including height, shape, and diameter, especially in combination with proper assisting templates^46,47^. Since the entire area covered by the PR could be coated with gold via ECD, after removing the PR nanohole templates, large-scale homogenous CAuNEs could be easily obtained. Here, CAuNEs of three different diameters were fabricated (diameter of each cylinder was 500 nm, 700 nm, or 900 nm) while the electrical current during deposition process was monitored (Supplementary Fig. 1). Although the diameter of each gold cylinder varied, the distance between each nanoelectrode was kept constant (300 nm). As shown in Fig. 2(b,c), both PR nanohole arrays and CAuNEs were successfully fabricated on the ITO electrode surface and their diameters perfectly matched with the expected diameters based on the following theoretical calculation.

$${\rm{\Lambda }}={{\rm{\lambda }}}_{{\rm{UV}}}/2\,\mathrm{Sin}\,{\rm{\theta }}$$

where Λ, λ_UV_, and θ are the pitch (nm), wavelength of the UV laser (360 nm), and the incidence angle (°), respectively. However, unlike the PR nanoholes, variations in the diameters of CAuNEs were within approximately 30%, which might be due to the difference in the diffusion of the gold chloride mixture solution used for ECD. It seemed that the parameters related to the concentrations of each component in the gold mixture solution (e.g., HAuCl_4_, surfactant, electrolyte) and the ECD conditions (e.g., deposition time, applied voltage) should be carefully tuned to overcome obstacles in nanohole-assisted CAuNE fabrication.

### Optimization of nanotopographic features of CAuNEs

**Figure 3:**
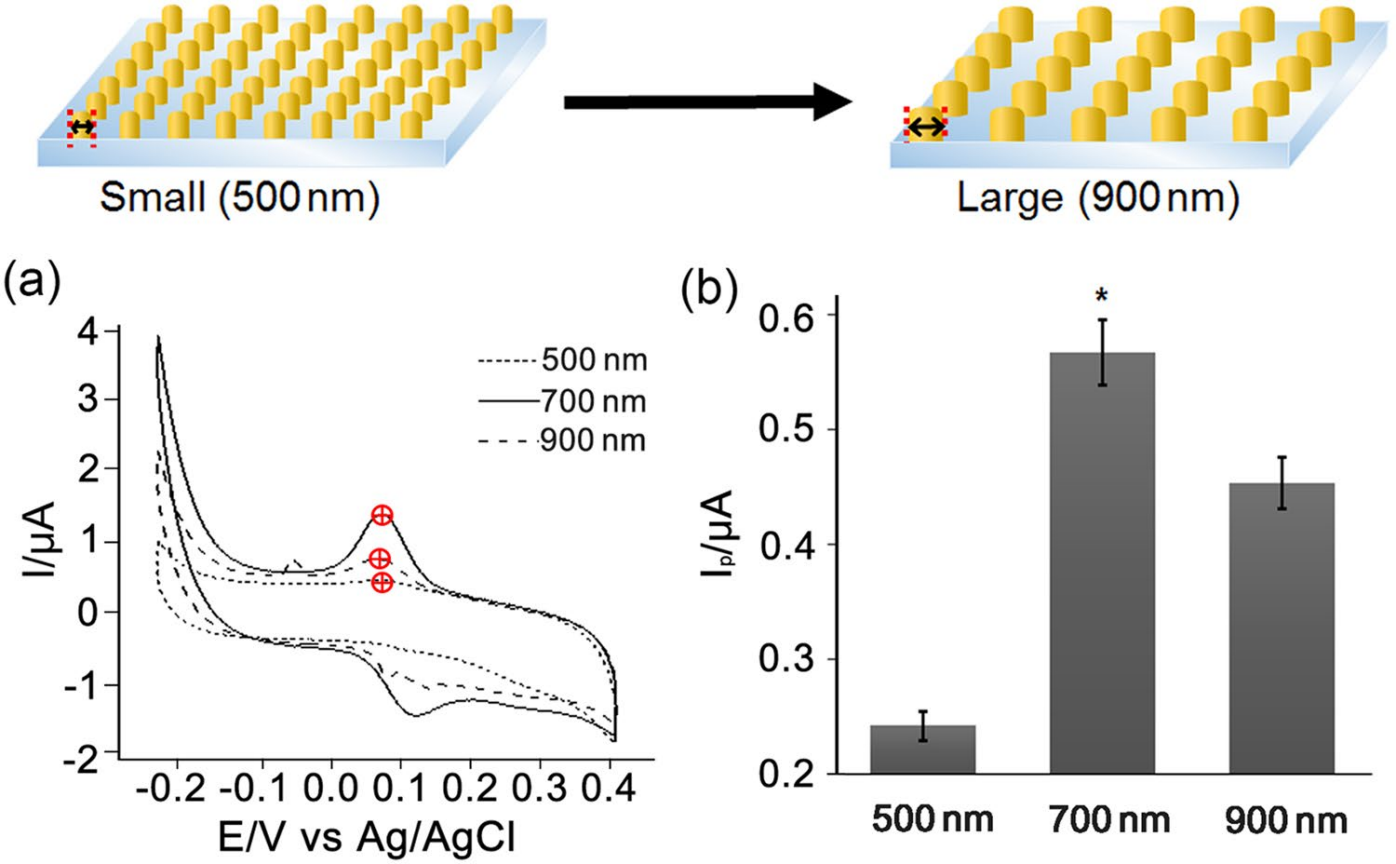
Optimization of nanopattern diameter for enhancement of the dopamine-specific electrochemical signals. (**a**) Schematic showing three different diameters of the CAuNEs. (**b**) CV signals of each fabricated nanoelectrode. (**c**) Calculated peak intensities from CV signals of (**b**) presented as a bar graph (N = 3). *p < 0.05.

CV was performed to investigate the effect of the diameter of CAuNEs on the ECD of dopamine, and the redox peaks achieved for each CAuNE were compared. Considering that the density of the nanoelectrode was critical in enhancing its sensitivity, mostly owing to the surface-to-volume ratio, we hypothesized that the CAuNE with a diameter of 500 nm would be the best for dopamine detection. To confirm this hypothesis, the fabricated CAuNEs with diameters of 500 nm, 700 nm, and 900 nm were analyzed by CV in the presence of 10 µM dopamine (PBS; pH = 7.4). As shown in Fig. 3(a), all the substrates showed dopamine-specific electrochemical signals, which were around 0.09 V and 0.14 V for the reduction and oxidation peaks, respectively. Specifically, the peak-to-peak separations, |E_pa_ − E_pc_| values, were 0.044 V, 0.041 V, and 0.036 V, while the I_pa_/I_pc_ values were 1.48, 1.44, and 1.58 for CAuNEs with diameters of 500 nm, 700 nm and 900 nm, respectively. Consequently, the calculated values indicated that the reduction and oxidation of dopamine on the electrode, that is, the forward and backward reaction of dopamine to dopamine-o-quinone, was quasi-reversible. Remarkably, based on the reduction peaks (I_pc_) marked as red circles in Fig. 3(a), CAuNE-700 nm showed a stronger signal than CAuNE-500 nm and CAuNE-900 nm (0.242 µA, 0.561 µA, and 0.449 µA for 500 nm, 700 nm, 900 nm, respectively). Specifically, the I_pc_ value obtained for CAuNE-700 nm was 2.33 and 1.24 times higher than that for CAuNE-500 nm and CAuNE-900 nm, respectively. The CAuNE-700 nm platform showed that the conditions to achieve the highest dopamine redox signal were clearly different from those hypothesized by us, that is, the CAuNE with the smallest diameter (500 nm) would be the best for the detection of the target analyte. This could be partially explained by the small diameter of the PR nanoholes (500 nm), which make the gold solution difficult to diffuse at the bottom of the ITO electrode surface, thus ultimately resulting in a decrease and/or failure in the gold deposition in each nanohole array. Taken together, we confirmed that the CAuNE-700 nm platform was the best for electrochemical detection of dopamine and was suitable for further study, including the sensitivity and selectivity tests.

### Electrochemical detection of dopamine using various heights of CAuNEs

**Figure 4:**
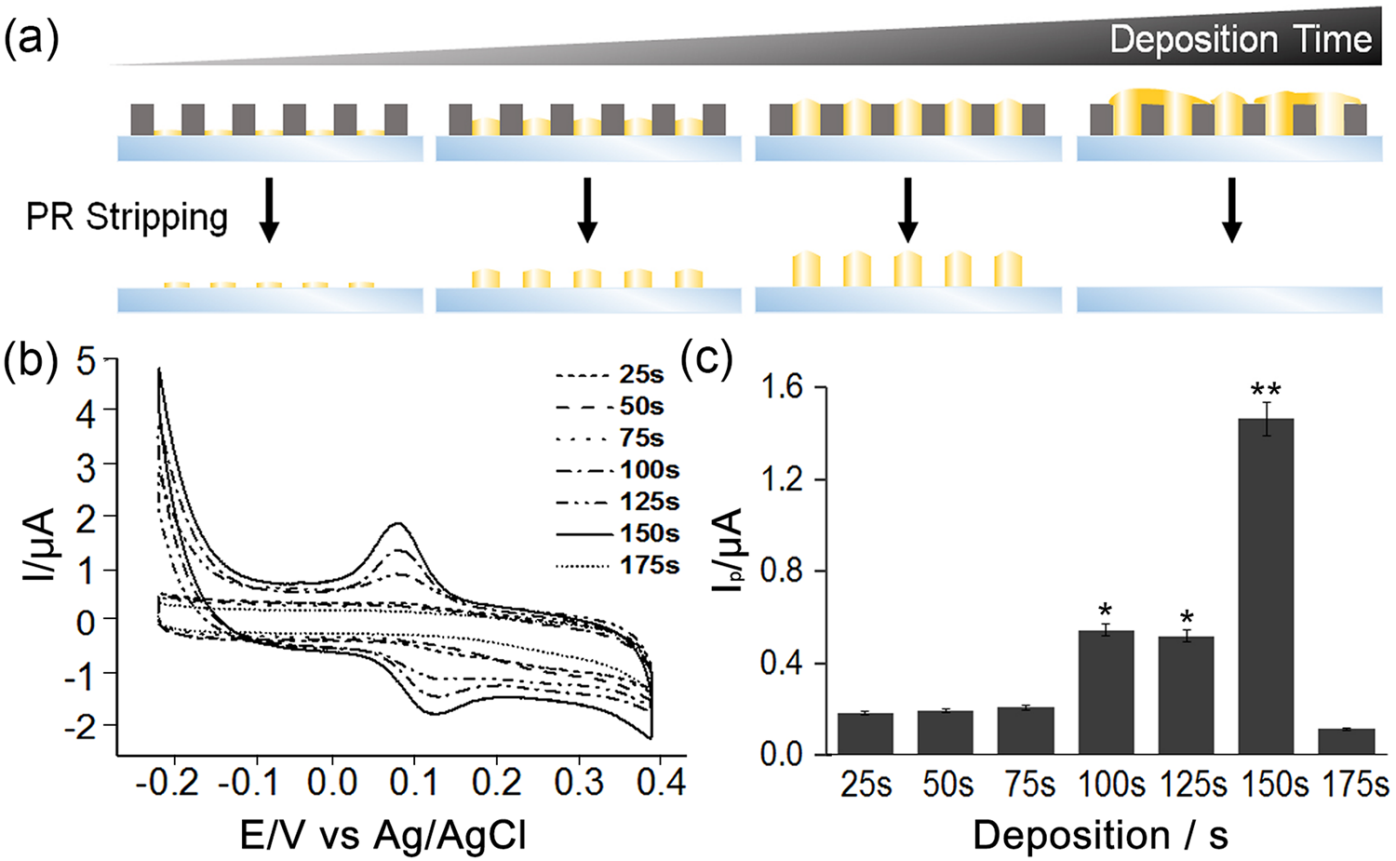
Gold deposition time optimization. (**a**) Schematic shows that the PR removal step and differences in results depending on the deposition time. (**b**) CV signals for different deposition times, 25, 50, 75, 100, 150, and 175 s. The nanoelectrode with a deposition time of 150 s showed the strongest signal. (**c**) Calculated peak intensities from (**b**) presented as a bar graph (N = 3). *p < 0.05 and **p < 0.01.

After optimizing the diameter of the nanocylinders for dopamine detection, we next sought to investigate the maximum height of the nanocylinders formed by ECD, which would ultimately affect the sensitivity of the CAuNE platform. It is certain that the nanocylinder height is a critical factor to enhance the dopamine redox signals and should thus reach its maximum limit to increase the surface area of the nanocylinders. However, owing to several structural limitations of the nanocylinders, such as the height limitation on the PR nanohole template and the aspect ratio of the nanocylinders, which are strongly related to their structural stability, the average height of the nanocylinders needs to be optimized, preferably based on the redox signal intensities at a fixed concentration of dopamine. To this end, as shown in Fig. 4(a), the time needed for ECD was varied from 25 to 175 s to achieve nanocylinder arrays of different heights on the ITO electrode surface. While varying the height of the nanocylinders, their diameter was fixed at 700 nm and the distance between each cylinder was maintained at 300 nm. Figure 4(b) shows the CV profiles of each CAuNE with different ECD times in the presence of 10 µM dopamine. Interestingly, among all the substrates fabricated, the best signal intensities were obtained for CAuNEs with a deposition time of 150 s (1.434 µA), which was 8.2, 7.8, 7.2, 2.7, 2.8, and 13.4 times higher than for CAuNEs with deposition times of 25 (0.175 µA), 50 (0.185 µA), 75 (0.198 µA), 100 (0.526 µA), 125 (0.504 µA), and 175 s (0.107 µA), respectively (Fig. 4(c)). For CAuNEs with a deposition time of 175 s, the overgrowth of gold nanocylinders was observed, which resulted in the complete detachment of nanocylinders during the removal of the PR nanohole template. In this case, the gold nanocylinder-mediated redox reactions of dopamine could not occur, which was a clear rationale for the large decrease in reduction current (Ipc) of CAuNEs with a deposition time of 175 s. Next, both the heights profile of the fabricated electrodes and active electrode surface area were investigated. The active surface area of CAuNE with 150 s deposition time, which showed the best performance for the dopamine detection, was calculated to be 0.271 cm^2^ based on the cyclic voltammogram obtained using K_3_Fe(CN)_6_ as a redox couple (Supplementary Fig. 2). The height of CAuNE with 150 s deposition time was confirmed to be 186 nm while the same substrates with 25 s, 50 s, and 100 s deposition time were measured to be 49.292 nm, 100.4 nm and 127.067 nm, respectively, based on the atomic force microscopic images (Supplementary Fig. 3). This finding proves that the increase of deposition time actually increased the heights of CAuNEs, which resulted in the enhancement of electrochemical signals of dopamine. Hence, it could be concluded that considering the structural stability and maximum height of the nanocylinders, which was fabricated by sequential LIL, ECD, and nanohole removal processes, nanocylinders with an ECD time of 150 s (186 nm in height), a diameter of 700 nm, and a gap of 300 nm were the best for the sensitive electrochemical detection of dopamine.

### Detection of dopamine using CAuNEs

**Figure 5:**
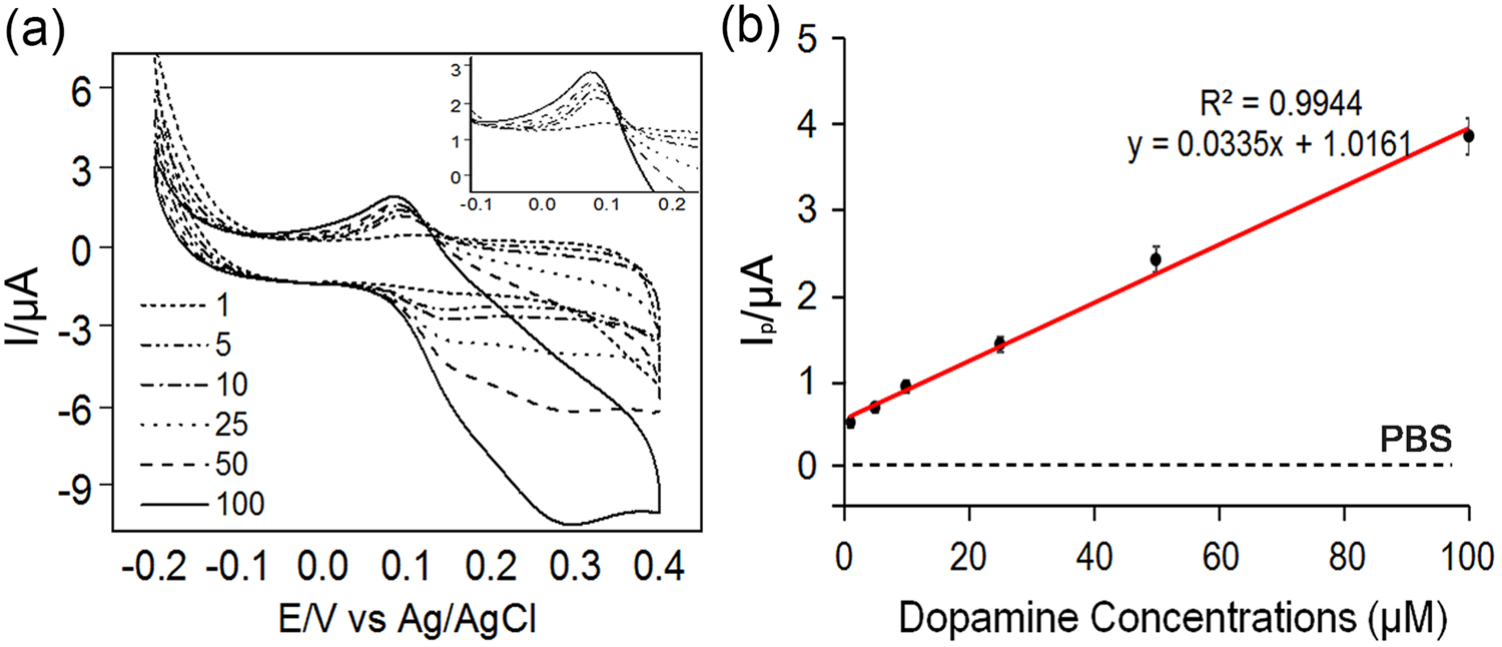
Linear correlations between CV signals and dopamine concentration. (**a**) Changes in CV signals with varying concentrations of dopamine, from 1 to 100 µM. (**b**) Linear correlation (R^2^) between dopamine concentration and intensity of the reduction current calculated from the CV signals.

To confirm the potential of the newly developed platform for practical sensing applications, the following critical factors must be considered: (1) highly linear correlation between the signal intensity and the concentration of the target analyte (R^2^ > 0.99) and (2) high selectivity toward the target molecule in the presence of other interfering molecules. First, the reduction peaks for varying concentrations of dopamine were obtained. As shown in Fig. 5(a), stable reduction and oxidation peaks of dopamine were observed in the CV curves for the CAuNE-700 nm platform with varying dopamine concentrations. No dopamine-specific redox signals (E_pc_ = 0.09 V, E_pa_ = 0.14 V vs. Ag/AgCl) were observed in the absence of dopamine (PBS only), and the background signal was found to be stable and constant for more than three cycles. Based on the reduction peaks (I_pc_), the CAuNE-700 nm platform showed good performance in terms of dopamine detection, with a linear dopamine concentration range of 1–100 µM (R^2^ = 0.9944). The limit of detection (LOD) of the CAuNE-700 nm platform was found to be 5.83 µM based on the calculated parameters, the slope of the calibration curve, and the standard deviation of the response.

**Figure 6:**
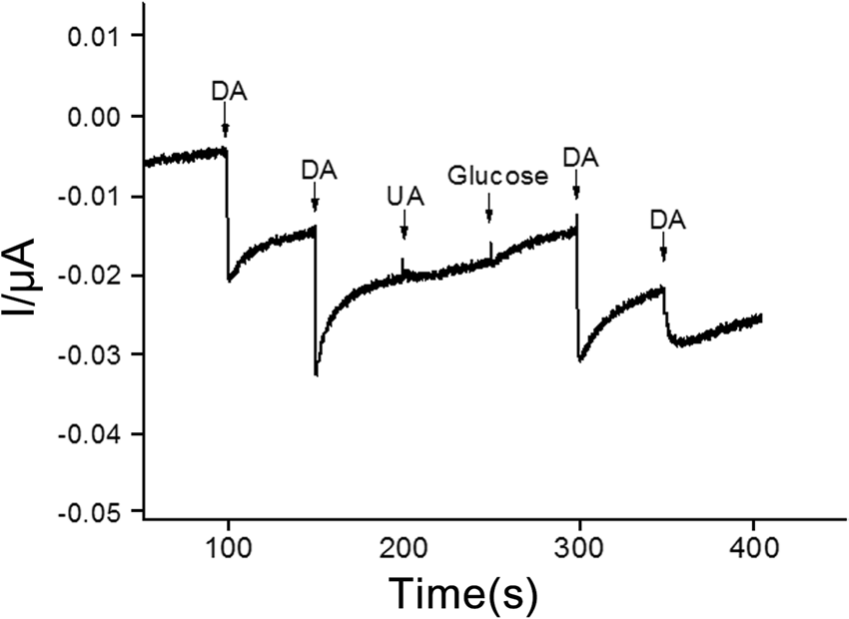
Selectivity testing using amperometric detection of 10 µM dopamine with two interfering molecules, UA and glucose; PBS (100 mM, pH = 7.0) was used as the electrolyte. Applied potential was 0.3 V vs. Ag/AgCl.

After confirming the linearity, the selectivity of the CAuNE-700 nm platform toward dopamine was evaluated. Since the dopamine co-exists with various biomolecules such as UA, AA, glucose, and proteins in human blood plasma, it is important to confirm the ability of the platform for the selective detection of dopamine^19,30^. Here, we selected UA and glucose as the interfering molecules for amperometric detection of dopamine using the CAuNE-700 nm platform. As shown in Fig. 6, the changes in the amperometric current (16.3–17.9 nA) were evident with the addition of 10 µM dopamine, while no changes were observable with the addition of the same concentration of UA and glucose. Remarkably, the additional dopamine treatment on the CAuNE-700 nm platform right after the addition of UA and glucose caused significant changes in the amperometric current (16.7 nA), and the electrical response of the platform was almost the same as that observed without UA and glucose. Remarkably, the dopamine was also detectable in a quantitative manner within the range of 1–100 μM in the presence of glucose (40 g/l), uric acid (44 mM) and human serum albumin (0.1 g/l) that is similar to the composition of real human plasma (Supplementary Fig. 4). Taken together, we can conclude that the developed CAuNE electrode is an outstanding platform for the detection of dopamine in highly sensitive and selective manner, even for the real human plasma sample.

### Cell cultivation and detection of dopamine on CAuNEs

**Figure 7:**
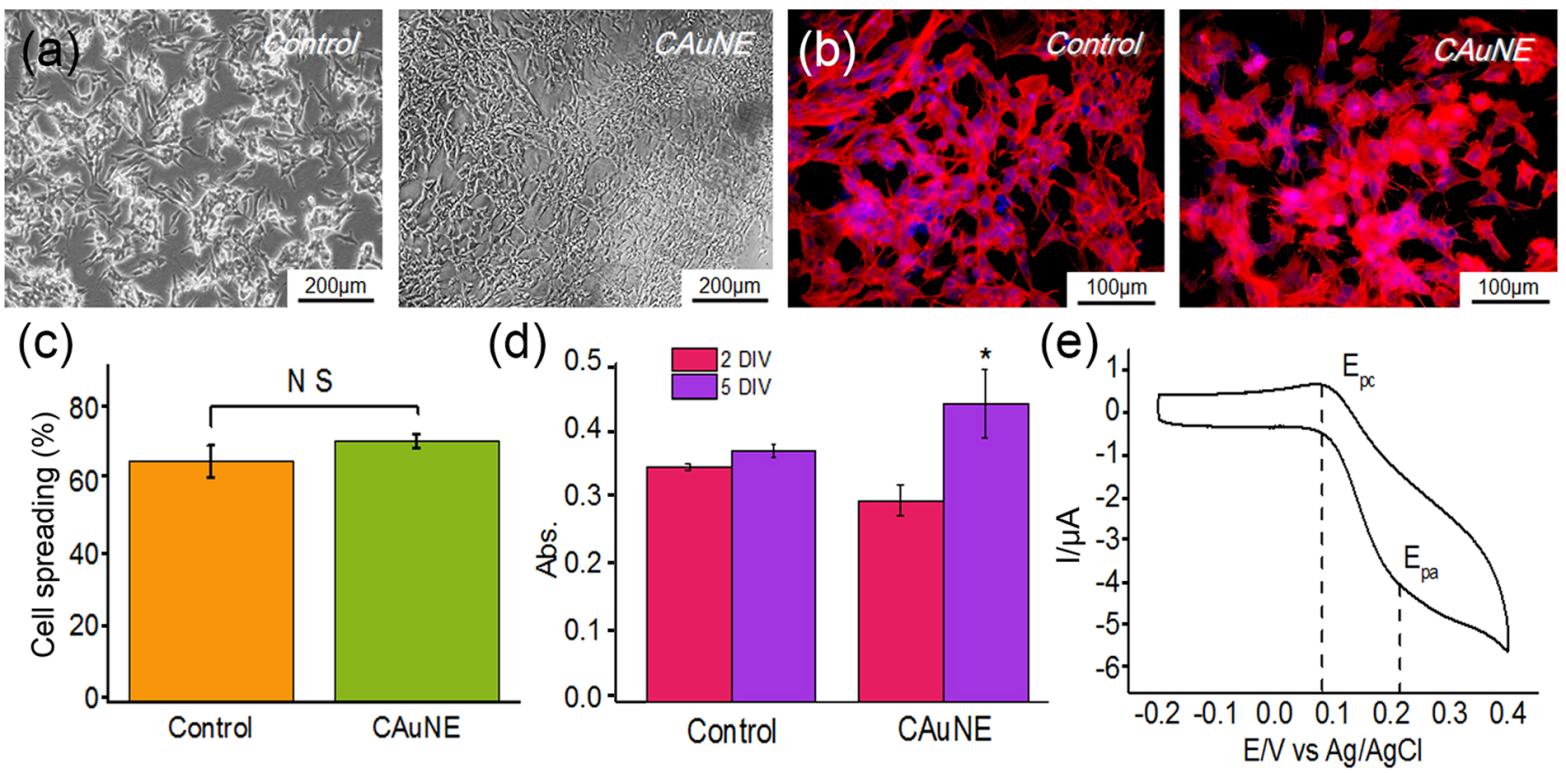
(**a**) Optical microscopic images of SH-SY5Y cells on the CAuNE-700 nm platform and the TCP. (**b**) Fluorescence microscopy images of F-actin-stained SH-SY5Y cells cultured on the CAuNE platform and the TCP. (**c**) Quantification graph of cell spreading area. (**d**) Cell viability test for 2 DIV and 5 DIV. *p < 0.05 (**e**) CV signals of dopamine in SH-SY5Y cells on the CAuNE platform.

Next, we aimed to use CAuNEs as real-time cell-based biosensors that are different from numerous previously reported typical dopamine sensors. Specifically, cells are known to actively interact with their surroundings via binding events between cell adhesion receptors such as integrins and ECM proteins^48^. Interestingly, many studies reported that nanopatterns generated on the underlying substrates affect several aspects of cell behavior and cell functions, including migration, proliferation, adhesion, spreading, and differentiation. Interestingly, among the various nanopatterns, homogeneous nanostructures were found to enhance cell adhesion on the substrate via integrin clustering, which ultimately resulted in changes in the cytoskeletal dynamics of the target cells^49–52^. Since the CAuNE-700 nm platform was composed of homogeneous periodic nanocylinder arrays, we hypothesized that this homogeneous gold electrode array would be beneficial to attach neuronal cells that secrete neurotransmitters as well as to achieve highly sensitive dopamine signals. To achieve this goal, the adhesion and growth of a representative human neuronal cell, SH-SY5Y, were assessed. SH-SY5Y is a human cell line derived from SK-N-SH line and has been identified as a neuronal cell expressing several dopaminergic markers. Hence, SH-SY5Y is a good *in vitro* model for testing the effects of chemical drugs/proteins on the dopamine secretion of dopaminergic neurons as well as for evaluating the efficacy of newly developed anticancer drugs on neuroblastoma ablation^53^. Thus, we first analyzed the adhesion and growth of SH-SY5Y to test the potential of the CAuNE-700 nm platform as a multifunctional platform enabling both cell cultivation of human dopaminergic neurons and dopamine detection. As shown in Fig. 7(a,b), the cytoskeletal arrangement of SH-SY5Y were found to be similar as confirmed by F-actin-stained fluorescence images; however, there were slight differences in cell spreading on the CAuNE-700 nm platform and the tissue culture plate (TCP) (72 µm^2^/cells and 66 µm^2^/cells, respectively, Fig. 7(c)). In the case of cell growth on the platform, based on the CCK-8 analysis, the SH-SY5Y cells showed a 6% higher proliferation rate at 5 days *in vitro* (DIV), while there was no remarkable difference in cell growth at 2 DIV. Remarkably, as a conducting cell culturing platform, a clear peak was observed in the voltammogram (I_p_ = 1.773 µA) with the addition of 100 μM dopamine in the presence of SH-SY5Y cells (3 DIV). Furthermore, to confirm the ability of the platform to measure the dopamine released from cells, the SH-SY5Y cells on the electrode surface were cultured with the medium containing 100 μM L-DOPA, a precursor of dopamine, for 2 hours and were triggered to release the dopamine with the addition of potassium chloride (120 mM KCl). As hypothesized, spike-like electrical signals were observed when the KCl solution was added to the cells (Supplementary Fig. 5), proving that the fabricated platform is able to measure not only chemical dopamine in the presence of cells, but also the dopamine produced by human dopaminergic neurons (SH-SY5Y). Taken together, it could be logically concluded that the developed CAuNE-700 nm platform was highly promising for both culturing human dopaminergic cells and detecting their neurotransmitter secretion *in vitro*, which might be useful for testing potential drugs for the treatment of various neurological diseases such as PD, ADHD, and drug addiction.

## Conclusion

Here, we reported a new dopamine detection platform composed of ITO and periodic gold nanocylinder arrays. By simply changing the incidence angles during interference lithography, homogeneous PR nanohole arrays of three different diameters (500 nm, 700 nm, 900 nm) were successfully formed on the surface of ITO. Gold nanocylinder arrays were then fabricated via ECD, and their dopamine detection ability was examined. According to the CV results, nanocylinder arrays with a diameter of 700 nm and a deposition time of 150 s (186 nm in height) showed the best performance for dopamine detection. The linear range of the CAuNE-700 nm platform was found to be 1–100 µM dopamine with an LOD of 5.83 µM^54^, which was suitable for the sensitive detection of neurotransmitters released from cells. Dopamine was also detectable in the presence of other biological molecules (e.g., glucose, UA, human serum albumin), proving the excellence of the developed platform for sensitive and selective dopamine detection. Moreover, owing to the periodic homogeneous feature of the CAuNE platform, which was known to be preferable for cell adhesion, human neuroblastoma cells (SH-SY5Y) were successfully cultured on the artificial electrode surface and showed enhanced cell growth at 5 DIV when compared with that on a conventional TCP. Further, both chemical dopamine (100 μM) and the dopamine release of cells with addition of KCl as a triggering molecule were detectable in presence of the SH-SY5Y cells at 3 DIV, proving the potential of the developed CAuNE platform for *in situ* label-free monitoring of dopamine released from neuronal cells.

In conclusion, our newly developed CAuNE platform is an excellent candidate for simple, convenient, and sensitive detection of dopamine and can be highly useful for early diagnosis of various neurological diseases (e.g., PD, schizophrenia, ADHD)^55^. The platform is also useful for other applications, including to test the function of dopaminergic neurons derived from various stem cell sources (e.g., embryonic stem cells, induced pluripotent stem cells, neural stem cells, mesenchymal stem cells) and for toxicity assessments of various chemicals and nanomaterials on human neuronal cells.

## Materials and Methods

### Commercial products and cells

ITO electrodes were purchased from U.I.D (Cheongju, Korea). All chemical materials used for LIL including hexamethyldisilazane (HMDS) and dimethyl sulfate (DMSO) were obtained from Sigma-Aldrich, and the PR (AZ nLof-2020), thinner (AZ 1500 thinner), and developer (AZ 300MIF developer) were obtained from AZ Electronic Materials, USA.

Commercial products for gold deposition including gold (III) chloride trihydrate and ammonium sulfate were purchased from Sigma-Aldrich, and tripotassium citrate monohydrate was purchased from Wako Pure Chemical, Ltd, Japan.

All materials used for electrochemical dopamine detection including dopamine hydrochloride and phosphate buffered saline (PBS) were obtained from Sigma-Aldrich. SH-SY5Y cells derived from human neuroblastoma were obtained from the Korean Cell Line Bank (KCLB). The cells were cultured in Dulbecco’s modified Eagle medium (DMEM)/nutrient mixture F-12 (F-12) obtained from Thermo Fisher Scientific in a ratio of 1:1.

A cell counting kit-8 (CCK-8) was purchased from Dojindo Laboratories for cell viability testing.

### Fabrication of homogenous polymer nanopatterns

The homogenous polymer patterns were obtained by laser interference photolithography using a Lloyd’s mirror interferometer. The interferometer generated light interference between light coming from a source (MXL-FN-360) and the reflected light from the mirror. First, ITO-coated glass (10 Ω/cm^2^, 0.5 mm thickness) was washed in 1% Triton X-100, deionized water, and 80% ethanol for 15 min and sonicated and dried using N_2_.

The washed ITO glass was coated with HMDS using spin coating and this HMDS-coated ITO glass was coated with the diluted PR and thinner (ratio of 6:4). This obtained substrate was then baked on a hot plate at 130 °C for 60 s (soft bake). The soft-baked substrate was exposed to UV (λ = 360 nm, 0.51 mW) using the Lloyd’s mirror interferometer. The substrate was exposed twice to UV to obtain PR nanohole patterns. The exposed substrate was then baked again under the same conditions as the soft bake (post-exposure bake). Subsequently, the substrate was treated in the developer to remove the unexposed PR and was washed with DI water. Further, the washed substrate was baked at 135 °C for 60 s (hard bake).

The PR patterns and CAuNE substrates were imaged by field emission scanning electron microscopy (FE-SEM, SIGMA, Zeiss, Germany) at an acceleration voltage of 10 kV.

### Cylindrical gold nanoelectrode fabrication

Before gold deposition, a plastic chamber was placed on the PR-nanopatterned ITO glass using polydimethylsiloxane mixed with a thermal curing agent (ratio of 10:1) as a glue. A solution containing 2 mM HAuCl_4_, 0.5 mM ammonium sulfate, and 1 mM tripotassium citrate monohydrate was used for the electrochemical gold deposition. The Ag/AgCl (1 M KCl) and a platinum wire was used as the reference and counter electrodes, respectively. Prior to the electrochemical deposition, the reduction and oxidation potentials of HAuCl_4_ on ITO electrode were first characterized by CV, as shown in the Supplementary Fig. 6. Gold could be deposited electrochemically via reduction of HAuCl_4_ as reported by previous study written as below^56^.

$${{\rm{AuCl}}}_{4\,({\rm{aq}}.)}^{-}+3{{\rm{e}}}^{-}={{\rm{Au}}}_{({\rm{s}})}+4{{\rm{Cl}}}_{({\rm{aq}}.)}^{-}$$

The deposition was performed using a DY2013 potentiostat (Digi-Ivy, Inc., Austin, TX) with −1.2 V potential for 25, 50, 75, 100, 125, 150, and 175 s. Then, the photoresist used as a template for gold deposition was chemically stripped for 60 s using DMSO as a solvent, followed by washing with DI water three times to remove residual DMSO. For calculation of the active surface area, the Randles-Sevcik equation was used described as,

$${i}_{p}=(2.69\times {10}^{5})\,{{\rm{n}}}^{3/2}{{\rm{AD}}}^{1/2}{{\rm{v}}}^{1/2}{\rm{C}}$$

where *i*_*p*_ = peak current, n = number of electrons involved, A = electrode area (m^2^), D = diffusion coefficient (m^2^/s), v = scan rate (V/s) and C = concentrations of analytes (mol/L).

The CAuNEs were characterized by scanning electron microscope (SEM) SIGMA (Carl Zeiss, Germany) and atomic force microscope (AFM) NX-10 (Park systems, Korea).

### Electrochemical detection

CV detection and amperometric detection were performed using a DY2013 potentiostat with 10 µM of dopamine hydrochloride solution diluted in PBS. The fabricated substrate consisting of the ITO electrode served as the working electrode. The reference electrode used was an Ag/AgCl (1 M KCl) electrode and a platinum wire was used as the counter electrode. For the CV analysis, dopamine hydrochloride solution in PBS was placed in the fabricated substrate chamber. The scan rate was 0.05 V/s, and the scanning range was −0.2 to 0.4 V.

For the dopamine selectivity test, amperometric detection was performed at a sampling time of 0.05 s and an initial voltage of 0.3 V, and the sensitivity was found to be 10^−6^ A. For electrochemical detection dopamine in the presence of SH-SY5Y, the medium for the cells grown for 3 days was removed and replaced with 100 µM dopamine hydrochloride solution diluted with PBS. For the detection of dopamine release from cells, L-Dopa (100 µM) dissolved in DMEM/F12 was treated for 2 hours prior to the amperometric detection and 120 mM KCl was added to trigger dopamine release from cells. Subsequently, electrochemical detection was analyzed under the same conditions as the preceding conditions. All of the experiments were performed at a temperature of 25 °C.

### Statistical analysis

The intensity of the cathodic peaks (I_pc_) detected by CV was quantitatively analyzed using the statistical program Origin 8 or Microsoft Excel 2013. Graphical data were presented as mean ± SE (N = 3). Statistical significance between the groups was analyzed by the unpaired Student’s t-test. Cell spreading was analyzed using Image J software.

### Cell culturing and fluorescence imaging

The cells (SH-SY5Y) were cultured in DMEM/F12 on the fabricated substrate coated with 0.01% poly L-lysine solution (Sigma-Aldrich) for 3 days. After 3 days of incubation, the cells were washed with PBS (pH = 7.4) and fixed with 4% formaldehyde solution for 10 min. Then the fixed cells were treated with 1% Triton X-100 diluted with PBS for 10 min and washed twice with PBS. Subsequently, the cells were stained with Alexa Fluor® 568 Phalloidin for 30 min at room temperature. The cells were washed twice with PBS and stained with Hoechst 33342 (Thermo Scientific, Germany) for 10 min. Finally, fluorescence imaging of the actin- and nucleus-stained cells was performed using an Eclipse 80i (Nikon Japan) microscope.

## Electronic supplementary material

Supporting information

## References

[1] Byeongju K (2013). Highly selective and sensitive detection of neurotransmitters using receptor-modified single-walled carbon nanotube sensors. Nanot..

[2] Sivakumar P (2014). Simultaneous electrochemical detection of dopamine and uric acid over ceria supported three dimensional gold nanoclusters. Mater. Res. Express..

[3] Howell LL, Cunningham KA (2015). Serotonin 5-HT2 receptor interactions with dopamine function: Implications for therapeutics in cocaine use disorder. Pharmacol. Rev..

[4] Kaya C, Block ER, Sorkin A, Faeder JR, Bahar I (2015). Multi-Scale Spatial Simulations Reveal the Effect of Dopamine Transporter Localization on Dopamine Neurotransmission. BpJ..

[5] Meyyappan M (2015). Nano biosensors for neurochemical monitoring. Nano Converg..

[6] Sangamithirai D, Munusamy S, Narayanan V, Stephen A (2016). Fabrication of neurotransmitter dopamine electrochemical sensor based on poly(o-anisidine)/CNTs nanocomposite. Surf. Interf..

[7] Dhanasekaran T (2018). Recent advances in polymer supporting layered double hydroxides nanocomposite for electrochemical biosensors. Mater. Res. Express..

[8] Buddhala C (2015). Dopaminergic, serotonergic, and noradrenergic deficits in Parkinson disease. Ann. Clin. Transl. Neurol..

[9] Howes OD, McCutcheon R, Owen MJ, Murray RM (2017). The Role of Genes, Stress, and Dopamine in the Development of Schizophrenia. Biol. Psychiatry..

[10] Schulz-Schaeffer W (2015). Is Cell Death Primary or Secondary in the Pathophysiology of Idiopathic Parkinson’s Disease?. Biomolecules..

[11] Petzinger GM (2015). The Effects of Exercise on Dopamine Neurotransmission in Parkinson’s Disease: Targeting Neuroplasticity to Modulate Basal Ganglia Circuitry. Neural Plast..

[12] Sitte HH (2017). Dopamine and noradrenaline, but not serotonin, in the human claustrum are greatly reduced in patients with Parkinson’s disease: possible functional implications. Eur. J. Neurosci..

[13] Choi W-S (2017). Conditional deletion of Ndufs4 in dopaminergic neurons promotes Parkinson’s disease-like non-motor symptoms without loss of dopamine neurons. Sci. Rep..

[14] Yi X (2017). Palladium nanoparticles entrapped in a self-supporting nanoporous gold wire as sensitive dopamine biosensor. Sci. Rep..

[15] Gaurab D, Chao T, Shabnam S, Prabhu UA (2016). Enabling long term monitoring of dopamine using dimensionally stable ultrananocrystalline diamond microelectrodes. Mater. Res. Express..

[16] Hubbard KE (2010). Determination of dopamine, serotonin, and their metabolites in pediatric cerebrospinal fluid by isocratic high performance liquid chromatography coupled with electrochemical detection. Biomed. Chromatogr..

[17] Luo Y, Ma L, Zhang X, Liang A, Jiang Z (2015). SERS Detection of Dopamine Using Label-Free Acridine Red as Molecular Probe in Reduced Graphene Oxide/Silver Nanotriangle Sol Substrate. Nanoscale Res. Lett..

[18] Purwidyantri A (2016). Spin-coated Au-nanohole arrays engineered by nanosphere lithography for a Staphylococcus aureus 16S rRNA electrochemical sensor. Biosens. Bioelectron..

[19] Sheng W (2017). Sensitive detection of dopamine via leucodopaminechrome on polyacrylic acid-coated ceria nanorods. Nanot..

[20] Ma J (2016). *In*–*situ* Molten Salt Template Strategy for Hierarchical 3D Porous Carbon from Palm Shells as Advanced Electrochemical Supercapacitors. ChemistrySelect..

[21] Rahman SF (2016). Selective determination of dopamine with an amperometric biosensor using electrochemically pretreated and activated carbon/tyrosinase/Nafion®-modified glassy carbon electrode. Biotechnol. Bioprocess Eng..

[22] Rand E (2013). A carbon nanofiber based biosensor for simultaneous detection of dopamine and serotonin in the presence of ascorbicacid. Biosens. Bioelectron..

[23] Sainio S (2015). Integrated Carbon Nanostructures for Detection of Neurotransmitters. Mol Neurobiol..

[24] Yu Y, Chen J, Zhou J (2014). Parallel-plate lab-on-a-chip based on digital microfluidics for on-chip electrochemical analysis. JMiMi..

[25] Chen PY, Vittal R, Nien PC, Ho KC (2009). Enhancing dopamine detection using a glassy carbon electrode modified with MWCNTs, quercetin, and Nafion. Biosens. Bioelectron..

[26] Wang Y, Li Y, Tang L, Lu J, Li J (2009). Application of graphene-modified electrode for selective detection of dopamine. Electrochem. Commun..

[27] Lin M (2015). A dopamine electrochemical sensor based on gold nanoparticles/over-oxidized polypyrrole nanotube composite arrays. RSC Advances..

[28] Mercante LA (2015). Electrospun Polyamide 6/Poly(allylamine hydrochloride) Nanofibers Functionalized with Carbon Nanotubes for Electrochemical Detection of Dopamine. ACS Appl. Mater. Interfaces..

[29] Qi S, Zhao B, Tang H, Jiang X (2015). Determination of ascorbic acid, dopamine, and uric acid by a novel electrochemical sensor based on pristine graphene. Electrochim. Acta..

[30] Choo SS (2017). Electrochemical Detection of Dopamine Using 3D Porous Graphene Oxide/Gold Nanoparticle Composites. Sensors (Basel)..

[31] Kim TH (2015). Large-Scale Nanoelectrode Arrays to Monitor the Dopaminergic Differentiation of Human Neural Stem Cells. Adv. Mater..

[32] Zetterström T, Sharp T, Marsden CA, Ungerstedt U (1983). *In Vivo* Measurement of Dopamine and Its Metabolites by Intracerebral Dialysis: Changes After d-Amphetamine. J. Neur..

[33] Wang J, Walcarius A (1996). Zeolite-modified carbon paste electrode for selective monitoring of dopamine. J. Electroanal. Chem..

[34] Bhalla N (2017). Plasma assisted large-scale nanoassembly of metal-insulator bioplasmonic mushrooms. ACS Appl. Mater. Interfaces..

[35] Bhalla N, Sathish S, Sinha A, Shen AQ (2018). Large‐Scale Nanophotonic Structures for Long‐Term Monitoring of Cell Proliferation. Adv Biosyst..

[36] Zhang X, Hicks EM, Zhao J, Schatz GC, Van Duyne RP (2005). Electrochemical Tuning of Silver Nanoparticles Fabricated by Nanosphere Lithography. Nano Lett..

[37] Musso NR, Vergassola C, Pende A, Lotti G (1989). Reversed-phase HPLC separation of plasma norepinephrine, epinephrine, and dopamine, with three-electrode coulometric detection. Clin. Chem..

[38] Valsecchi C (2016). Low-Cost Leukemic Serum Marker Screening Using Large Area Nanohole Arrays on Plastic Substrates. ACS Sensors..

[39] Weber de Menezes J, Thesing A, Valsecchi C, Armas LEG, Brolo AG (2015). Improving the performance of gold nanohole array biosensors by controlling the optical collimation conditions. ApOpt..

[40] Yu F (2005). Laser interference lithography as a new and efficient technique for micropatterning of biopolymer surface. Biomaterials..

[41] Xie Q (2008). Fabrication of nanostructures with laser interference lithography. J. Alloy. Compd..

[42] Stein K (2001). Fabrication of microsieves with sub-micron pore size by laser interference lithography. JMiMi..

[43] Chauvin A (2017). Large-Scale Fabrication of Porous Gold Nanowires via Laser Interference Lithography and Dealloying of Gold–Silver Nano-Alloys. Micromachines..

[44] Lee H-S (2018). Enhanced efficiency of crystalline Si solar cells based on kerfless-thin wafers with nanohole arrays. Sci. Rep..

[45] Ma D, Zhao Y, Zeng L (2017). Achieving unlimited recording length in interference lithography via broad-beam scanning exposure with self-referencing alignment. Sci. Rep..

[46] Gal D, Hodes G, Lincot D, Schock HW (2000). Electrochemical deposition of zinc oxide films from non-aqueous solution: a new buffer/window process for thin film solar cells. TSF..

[47] Hames Y, Alpaslan Z, Kösemen A, San SE, Yerli Y (2010). Electrochemically grown ZnO nanorods for hybrid solar cell applications. SoEn..

[48] Kafi MA, Cho H-Y, Choi J-W (2016). Engineered peptide-based nanobiomaterials for electrochemical cell chip. Nano Converg..

[49] Choi C-H (2009). Cell growth as a sheet on three-dimensional sharp-tip nanostructures. J. Biomed. Mater. Res. Part A..

[50] Choi C-H (2007). Cell interaction with three-dimensional sharp-tip nanotopography. Biomaterials..

[51] Martínez E, Engel E, Planell JA, Samitier J (2009). Effects of artificial micro- and nano-structured surfaces on cell behaviour. Ann. Anat.-Anat. Anz..

[52] Kang E-S (2017). Guiding osteogenesis of mesenchymal stem cells using carbon-based nanomaterials. Nano Converg..

[53] Yan X (2017). Protective effect of atmospheric pressure plasma on oxidative stress-induced neuronal injuries: an *in vitro* study. J. Phys. D: Appl. Phys..

[54] Armbruster DA, Pry T (2008). Limit of blank, limit of detection and limit of quantitation. Clin. Biochem. Rev..

[55] Suhito IR, Han Y, Min J, Son H, Kim TH (2018). In situ label-free monitoring of human adipose-derived mesenchymal stem cell differentiation into multiple lineages. Biomaterials..

[56] Hariri, M. B., Dolati, A. & Moakhar, R. S. The Potentiostatic Electrodeposition of Gold Nanowire/Nanotube in HAuCl_4_ Solutions Based on the Model of Recessed Cylindrical Ultramicroelectrode Array. *J. Electrochem. Soc.* **160**, D279–D288 (2013).

