# Supplementary material for: Electrochemical detection of dopamine using periodic cylindrical gold nanoelectrode arrays
Source: Sci Rep. 2018 Sep 19;8:14049. doi: 10.1038/s41598-018-32477-0 (PMC6145913; doi:10.1038/s41598-018-32477-0)
Supplement: Supplementary file 1 — Supporting information [file 41598_2018_32477_MOESM1_ESM.docx]

Supporting Information

Electrochemical detection of dopamine using periodic cylindrical gold nanoelectrode arrays

Da-Seul Kim^1^, Ee-Seul Kang^1^, Seungho Baek^1^, Sung-Sik Choo^1^, Yong-Ho Chung^2^, Donghyun Lee ^1^, Junhong Min^1,*^ and Tae-Hyung Kim^1, 3,*^

^1^ School of Integrative Engineering, Chung-Ang University, 84 Heukseok-ro, Dongjak-gu, Seoul 06974, Republic of Korea

^2^ Department of Chemical Engineering, Hoseo University, Asan City, Chungnam 31499, Republic of Korea

^3^ Integrative Research Center for Two-Dimensional Functional Materials, Institute of Interdisciplinary Convergence Research, Chung-Ang University, Seoul 06974, Republic of Korea

***** Correspondence: [junmin@cau.ac.kr](mailto:junmin@cau.ac.kr) (J.M.); [thkim0512@cau.ac.kr](mailto:thkim0512@cau.ac.kr) (T.-H.K.)


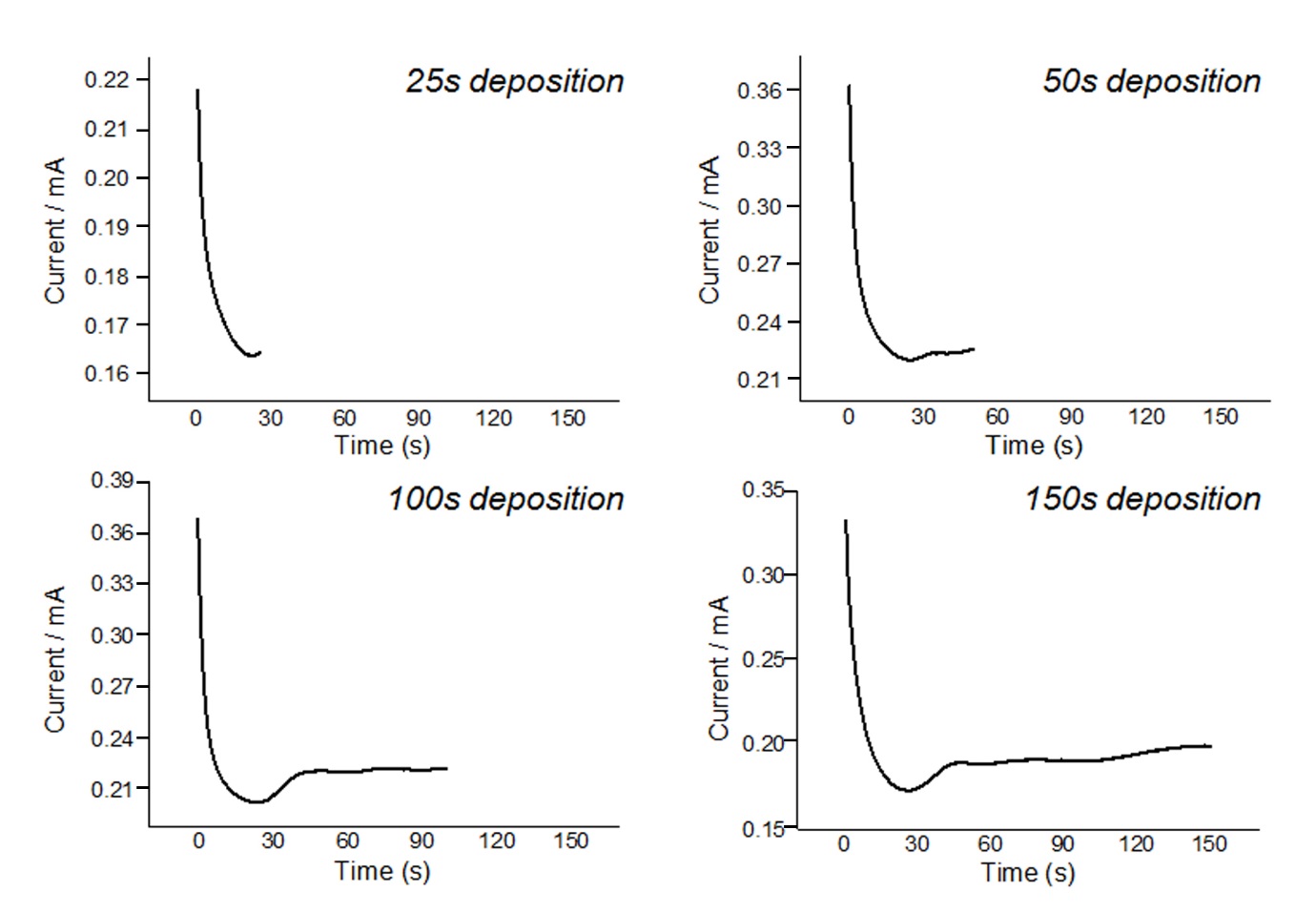


Supplementary Figure 1. Amperometric curves used for ECD of gold (25s, 50s, 100s, and 150s) through nanopatterned PR that was generated by LIL.


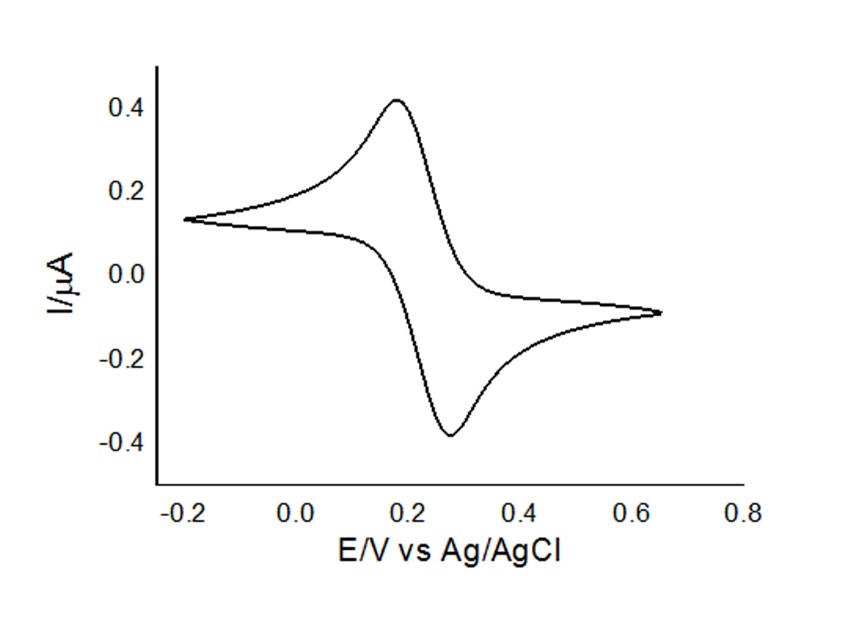


Supplementary Figure 2. Cyclic voltammetric curve using 10mM K_3_Fe(CN)_6_ in 1M KNO3 as an electrolyte for the calculation of active surface area of the 150s gold deposited electrode. Scan rate = 0.05 V/s.


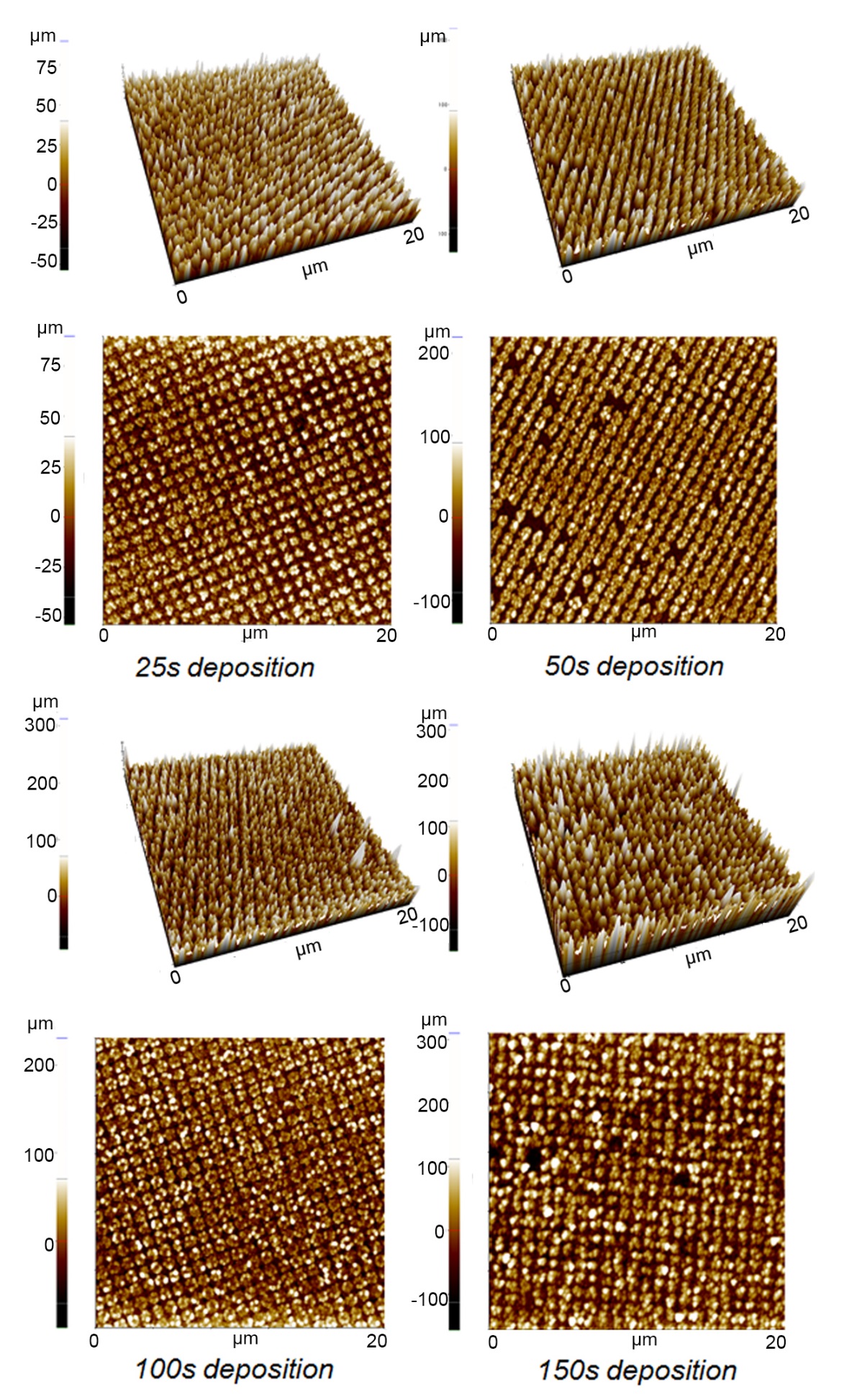


Supplementary Figure 3. The AFM images of CAuNEs with 25s, 50s, 100s, and 150s deposition time.


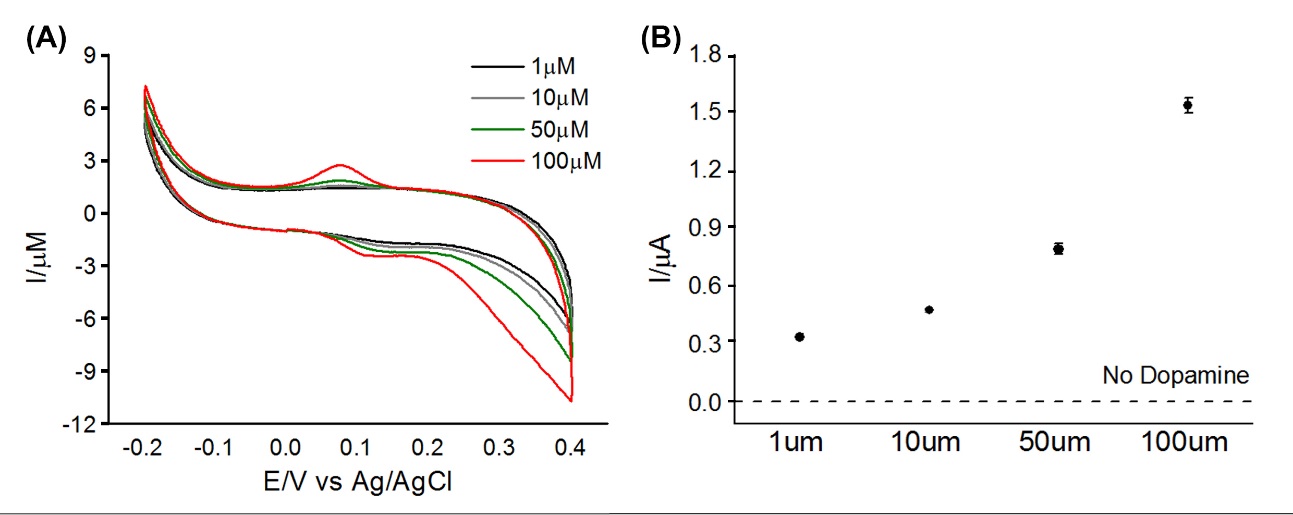


Supplementary Figure 4. (A) Cyclic voltammetric (CV) curves to measure different concentrations of dopamine (1µM, 10 µM, 50 µM, and 100 µM) in the presence of glucose (40g/l), uric acid (44mM), and human serum albumin (0.1g/l) as the interfering molecules.


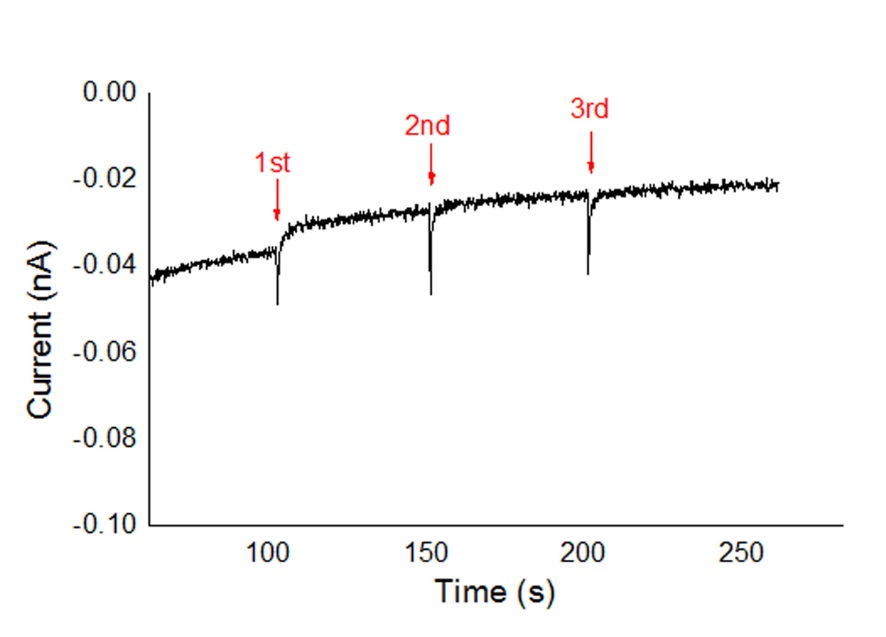


Supplementary Figure 5. Amperometric detection of dopamine release of SH-SY5Y wherein the dopamine was triggered 3 times by adding 120mM potassium chloride.


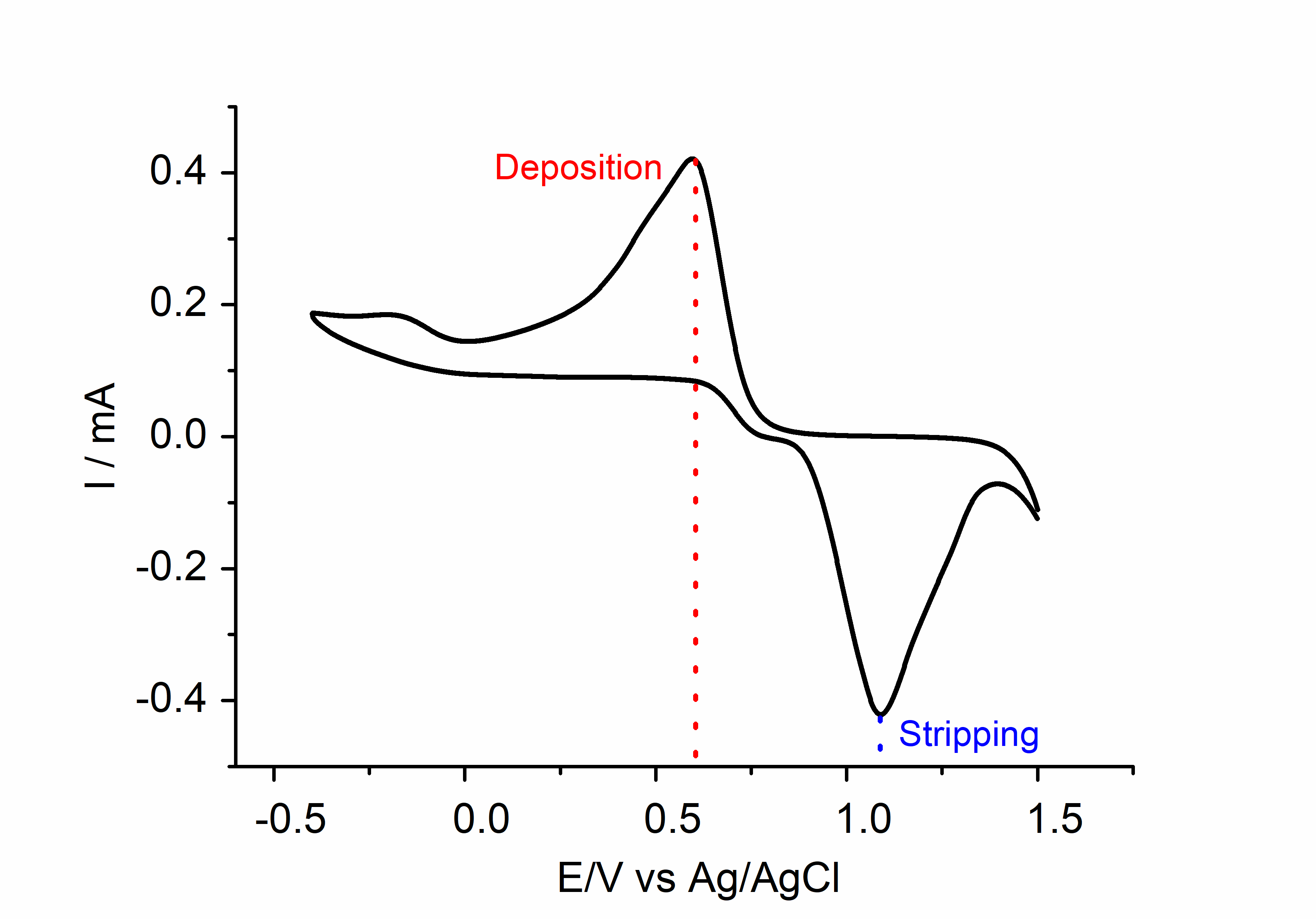


**Supplementary Figure 6.** Cyclic voltammetric (CV) curve of reduction and oxidation of HAuCl_4_ solution that was used as a precursor for the fabrication of gold nanocylinders.
